# Supplementary material for: Selective electrochemical oxidative coupling of methane mediated by Sr2Fe1.5Mo0.5O6-δ and its chemical stability
Source: Commun Chem. 2021 Sep 29;4:139. doi: 10.1038/s42004-021-00568-1 (PMC9814554; doi:10.1038/s42004-021-00568-1)
Supplement: Supplementary file 1 — Supplementary Information [file 42004_2021_568_MOESM1_ESM.pdf]

Selective electrochemical oxidative coupling of methane mediated by $Sr_2Fe_{1.5}Mo_{0.5}O_{6-\delta}$ and its chemical stability

Kannan P. Ramaiyan, Luke H. Denoyer, Angelica Benavidez, Fernando H. Garzon*

Chemical and Biological Engineering, University of New Mexico, Albuquerque, USA

Email: Garzon@unm.edu

Supplementary Note 1. General reactions involved in methane conversion towards ethylene.

Methane conversion to ethylene can occur through either OCM or NOCM processes. Reactions involving NOCM process is given in equations S1 and S2. The electrochemical redox reaction expected to occur at the electrode electrolyte interfaces during EC-OCM experiments for various reaction products are presented in equations S3 to S6.

Non-Oxidative Coupling of Methane (NOCM)

$$CH_4(g) = \frac{1}{2}C_2H_4(g) + H_2(g) \quad \text{(S1)}$$

$$CH_4 = C + 2H_2 \quad \text{(S2)}$$

Oxidative Coupling of Methane (OCM)

For Ethylene as product

$$2CH_4(g) + O^{2-} = C_2H_4(g) + H_2(g) + H_2O(g) + 2e^- \quad \text{(S3)}$$

$$2CH_4(g) + 2O^{2-} = C_2H_4(g) + 2H_2O(g) + 4e^- \quad \text{(S4)}$$

For CO as product

$$CH_4(g) + O^{2-} = CO(g) + 2H_2(g) + 2e^- \quad \text{(S5)}$$

$$CH_4(g) + 2O^{2-} = CO(g) + H_2(g) + H_2O(g) + 4e^- \quad \text{(S6)}$$

$$CH_4(g) + 3O^{2-} = CO(g) + 2H_2O(g) + 6e^- \quad \text{(S7)}$$

For CO_2 as product

$$CH_4(g) + 4O^{2-} = CO_2(g) + 2H_2O(g) + 8e^- \quad \text{(S8)}$$

Supplementary Note 2. Sr-O to $SrCO_3$ weight gain calculations

SFMO composition - $Sr_2Fe_{1.5}Mo_{0.5}O_{6-\delta}$ and SFMO molecular weight (MW) = 402.9 g/mol

SrO to $SrCO_3$ conversion results in an addition of two moles of CO_2 to SFMO's MW as two moles of Sr present in one more of SFMO (402.9+2*44) =490.97g/mol

Percentage weight gain by the addition of CO_2 = $(\frac{100}{402.9} * 490.97) = 121.83$

Thus, converting all of SrO in SFMO to $SrCO_3$ leads to a weight gain of 21.83% (assuming external oxygen supply).

Supplementary Table 1. Effect of flow rate dependence on the product distribution indicating a higher production of CO under reduced flow rates.

Potential	Flow rate	Peak 1a – Ethylene+CO	Peak 1b – H_2	Ratio (1b/1a)	Peak 2a – Ethylene+CO	Peak 2b – H_2	Ratio (2b/2a)
-0.5 V	50 SCCM	4.79	2.96	0.62	4.96	3.28	0.66
	75 SCCM	2.55	2.10	0.82	2.95	2.19	0.74
	100 SCCM	1.88	1.59	0.84	2.07	1.77	0.85
1.0 V	50 SCCM	11.72	5.02	0.43	6.35	5.98	0.94
	75 SCCM	6.84	3.29	0.48	3.59	3.77	1.05
	100 SCCM	4.52	2.71	0.60	2.26	2.93	1.30

Supplementary Table 2. Weight measurements on the SFMO-075Fe samples before and after TPO measurements in various CH_4 to O_2 ratio concentrations.

**Experiment gas concentration**	**SFMO-075Fe weight before exposure (grams)**	**SFMO-075Fe weight After exposure (grams)**	**Weight gain percentage**
100% CH_4	0.207	0.359	73.54
95% CH_4 : 5% O_2	0.206	0.414	101.33
90% CH_4 : 10% O_2	0.203	0.236	16.03

Supplementary Table 3. Quantification of XPS peaks associated with SFMO-075Fe samples before and after CH_4 treatment in TGA.

**Material**	**C1s %**	**Fe 2p %**	**Mo 3d %**	**O 1s %**	**Sr 3d %**
SFMO-075Fe as prepared	11.64	10.00	2.56	61.68	14.12
SFMO-075Fe CH_4 treated	76.50	0.68	0.67	16.38	5.78

Supplementary Figure 1

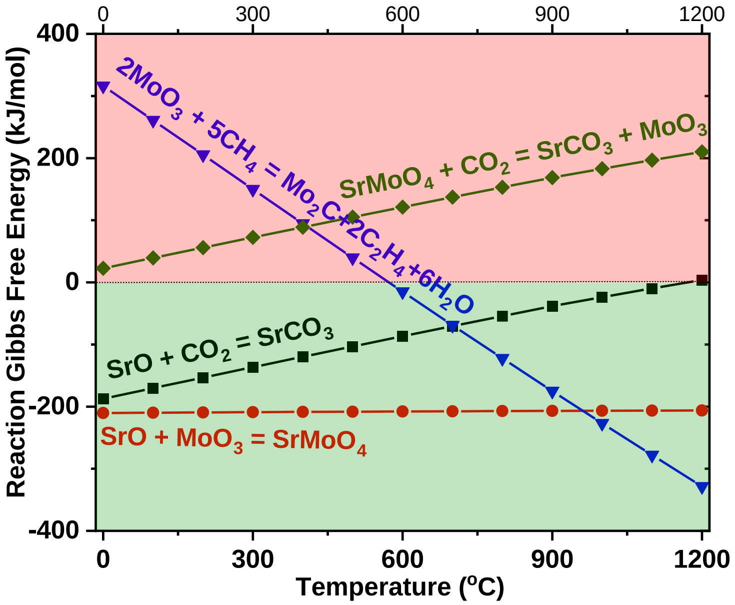

Supplementary Figure 1. HSC Chemistry calculations indicating the Gibbs reaction free energy change for some of the decomposition reactions involving the elements of SFMO.

Supplementary Figure 2

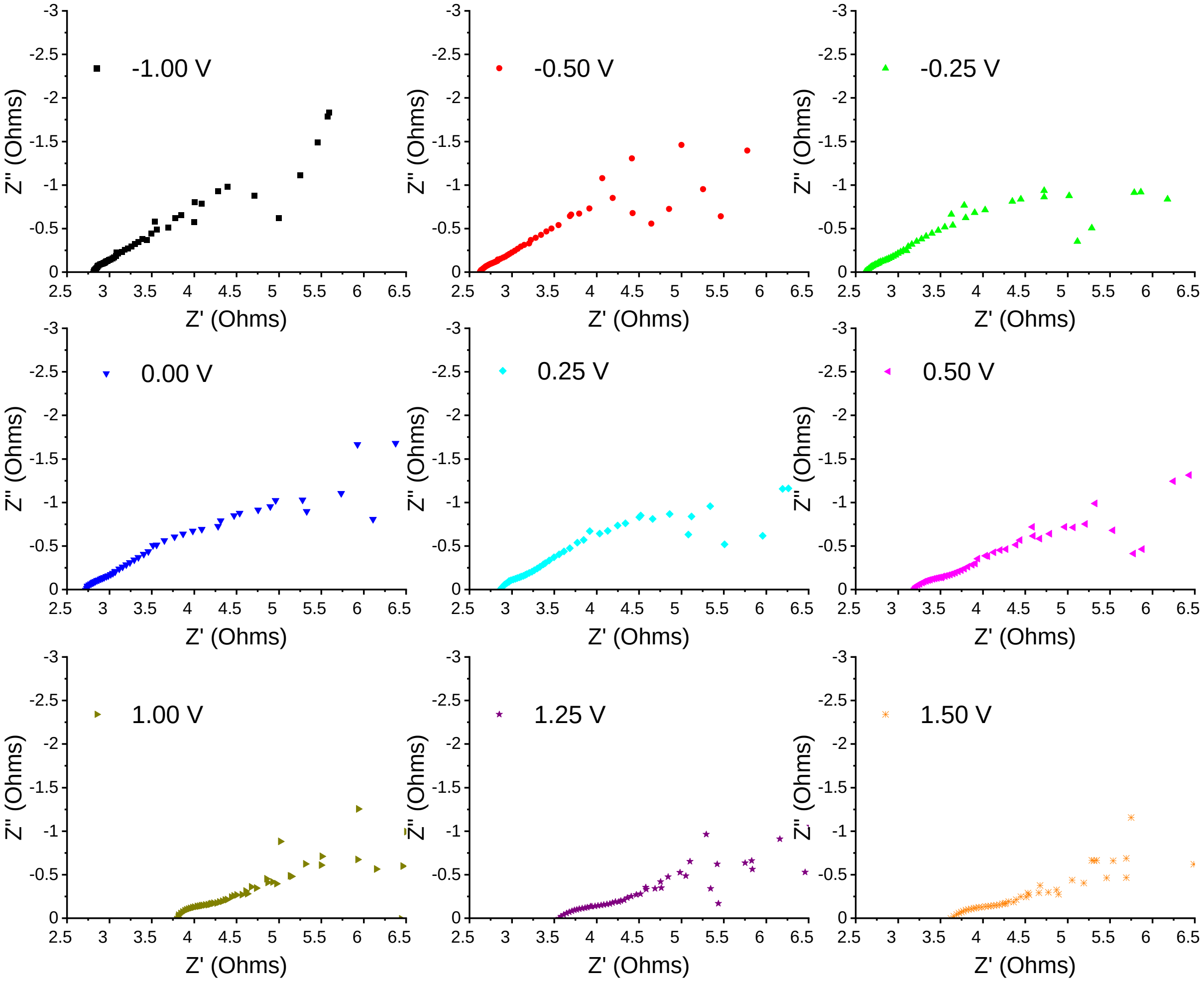

Supplementary Figure 2. Impedance Nyquist plots obtained at different bias conditions during the EC-OCM measurements with SFMO-075 catalyst.

Supplementary Figure 3

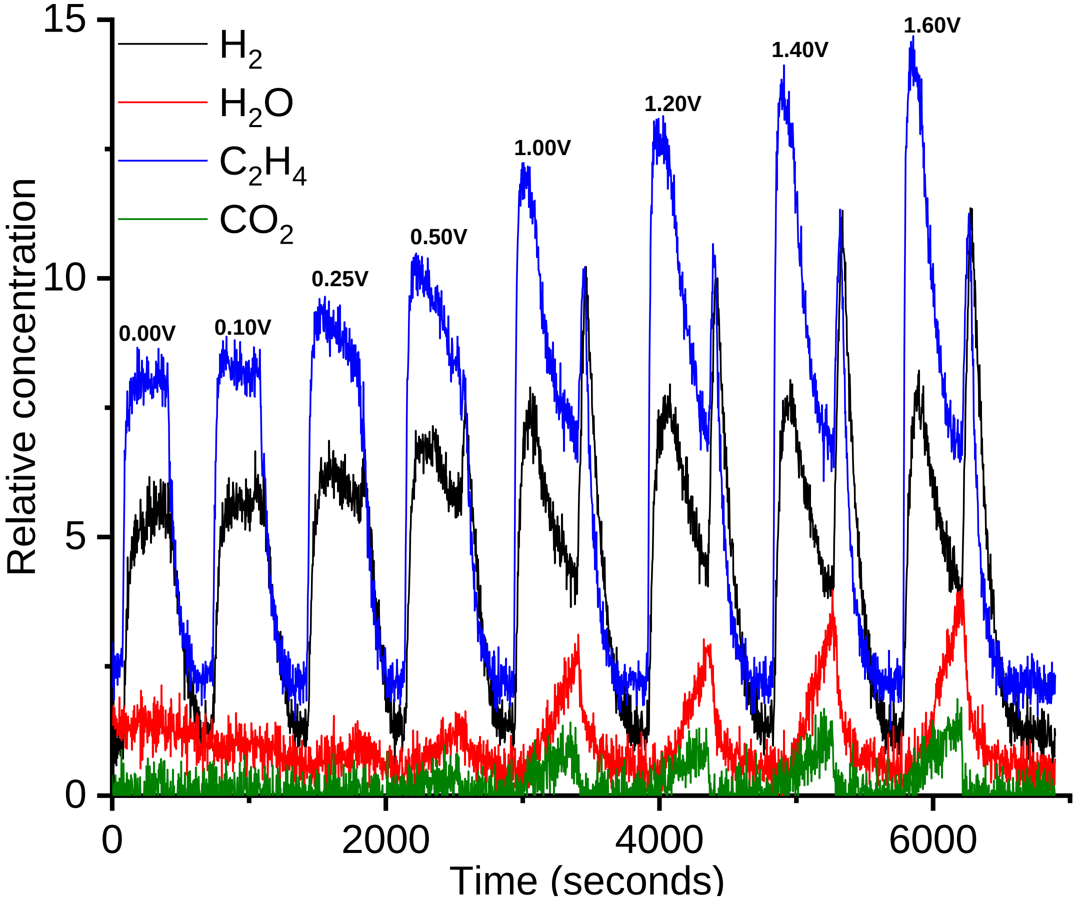

Supplementary Figure 3. Mass spec analysis of the EC-OCM measurements outlet stream during impedance measurements under different applied biases. The black color and red curves indicate H_2 and H_2O while blue and green curves indicate C_2H_4 and CO_2 respectively.

Supplementary Figure 4

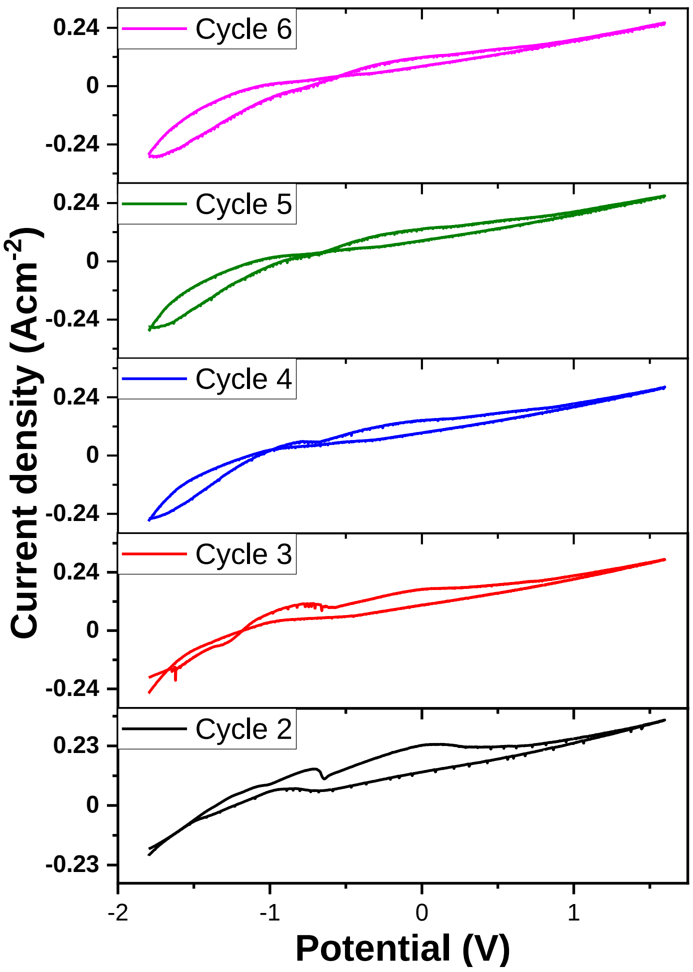

Supplementary Figure 4. Cyclic voltammetry result obtained with SFMO-075Fe during EC-OCM measurements at a scan rate of 1mV/s under pure methane in the anode and oxygen in the cathode at 850°C.

Supplementary Figure 5

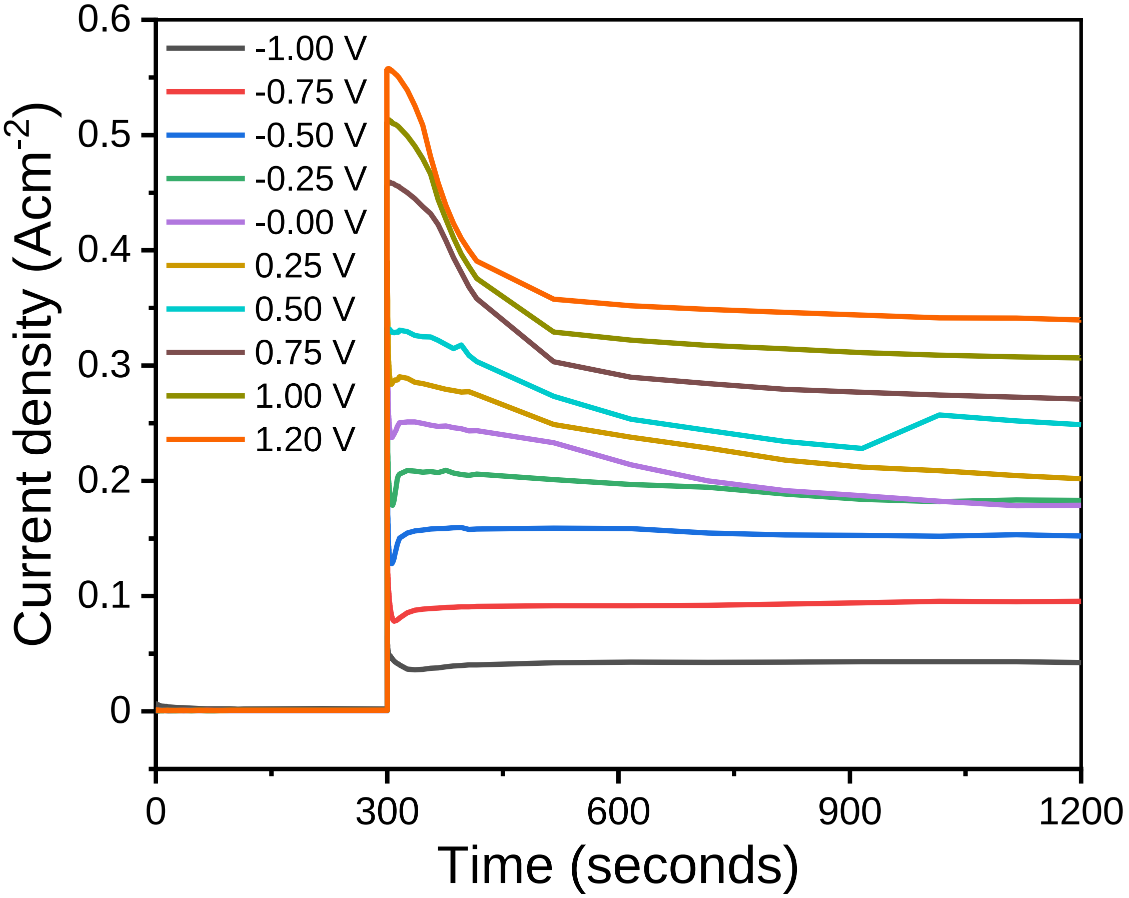

Supplementary Figure 5. Chronoamperometric plots obtained for the SFMO-075Fe electrode during EC-OCM measurements at various applied potentials from -1.00 V to 1.20 V.

Supplementary Figure 6

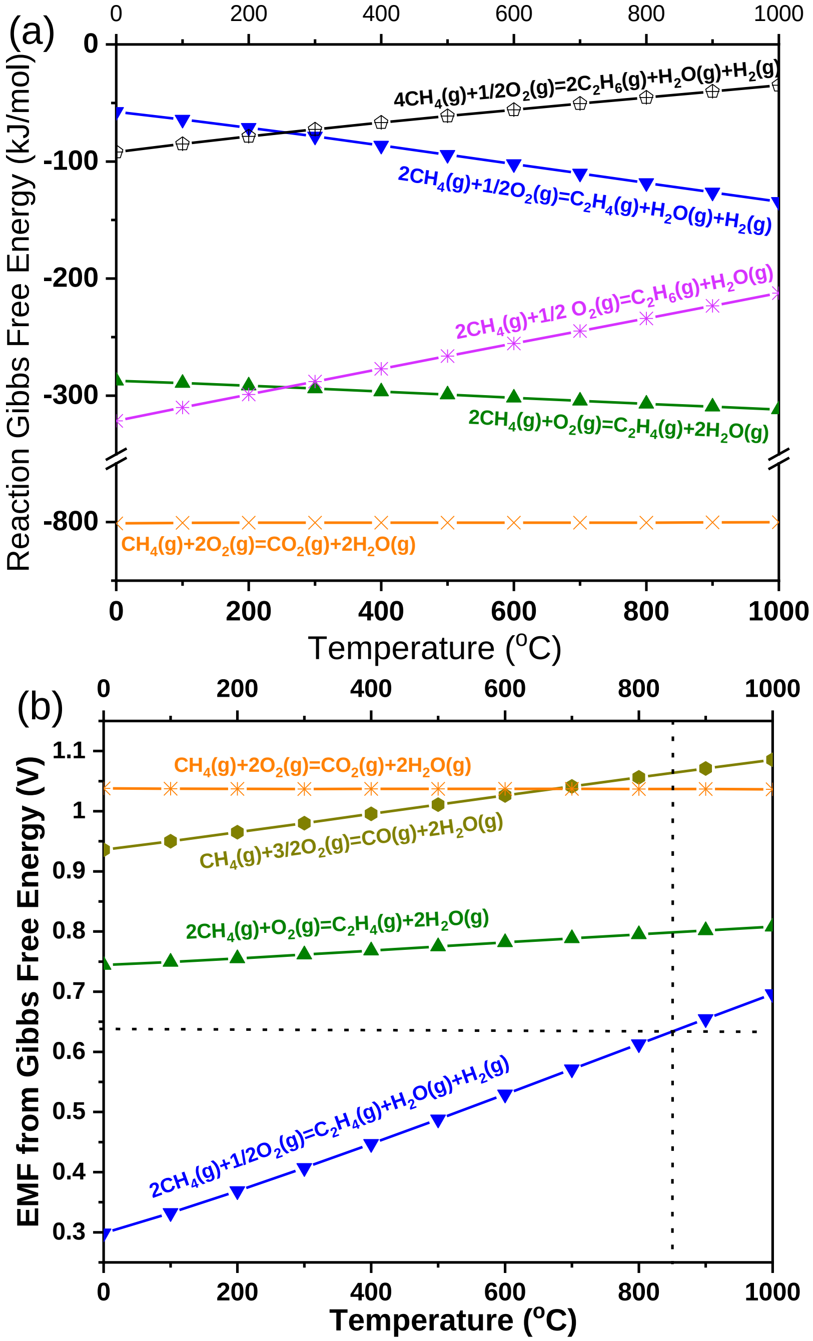

Supplementary Figure 6. HSC calculations on the (a) reaction Gibbs free energy for producing various oxidation products such as ethane, ethylene and carbon dioxide at various operating temperatures using CH_4 and O_2 as reactants, and (b) their corresponding EMF values calculated from the reaction Gibbs free energies.

Supplementary Figure 7

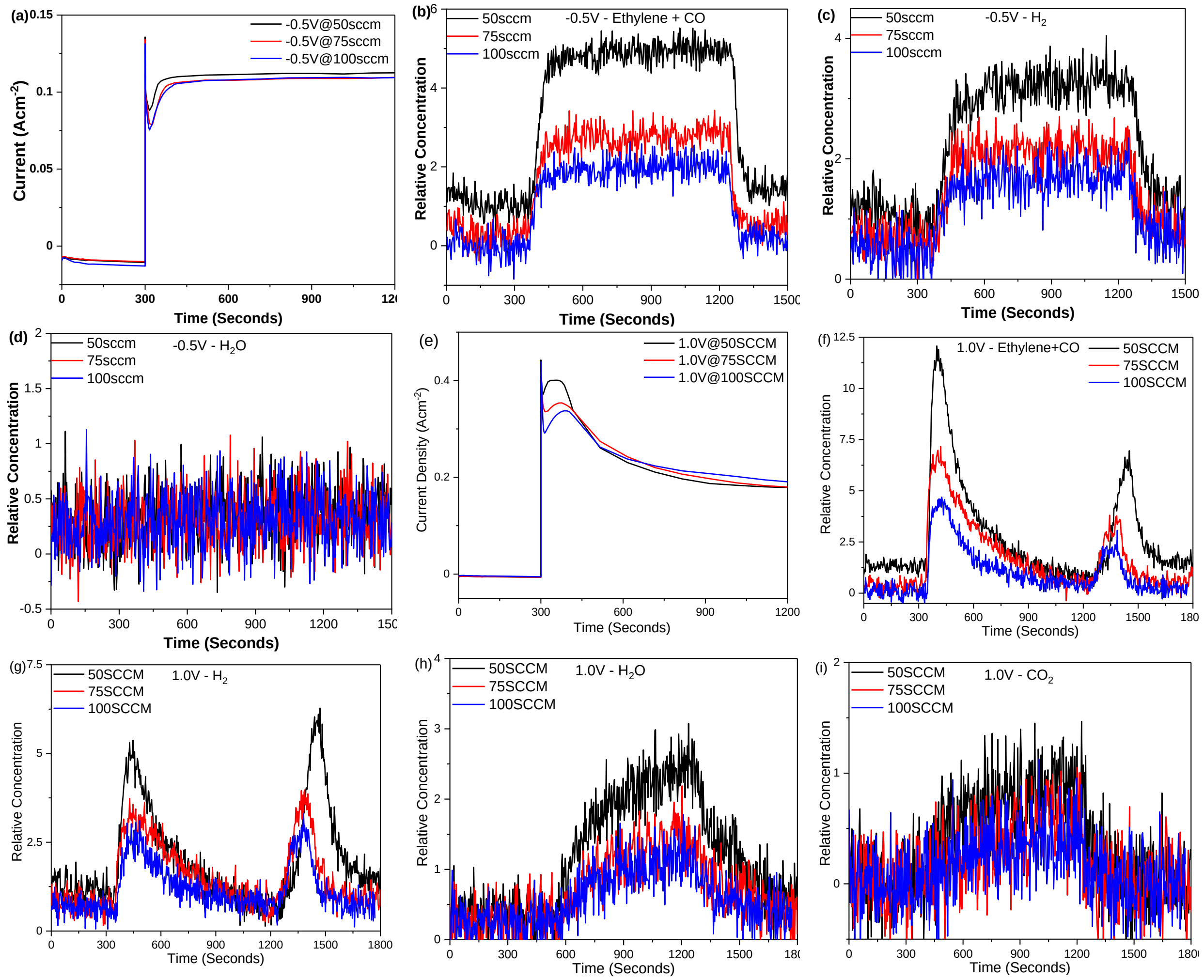

Supplementary Figure 7. (a) Chronoamperometric measurements carried out at a potential of -0.5V and at three different flow rates of 50, 75 and 100 SCCM. (b)-(d) corresponding mass spectroscopic analysis of the outlet stream. (e) CA measurements carried out at a potential of 1.0V and at three different flow rates of 50, 75 and 100 SCCM. Mass spectroscopic analysis of the outlet streams of 1.0V CA measurements for ethylene (f), hydrogen (g), water (h), and CO_2 (i). Black, red and blue curves in all figures indicate 50, 75, and 100 SCCM flow rates respectively.

Supplementary Figure 8

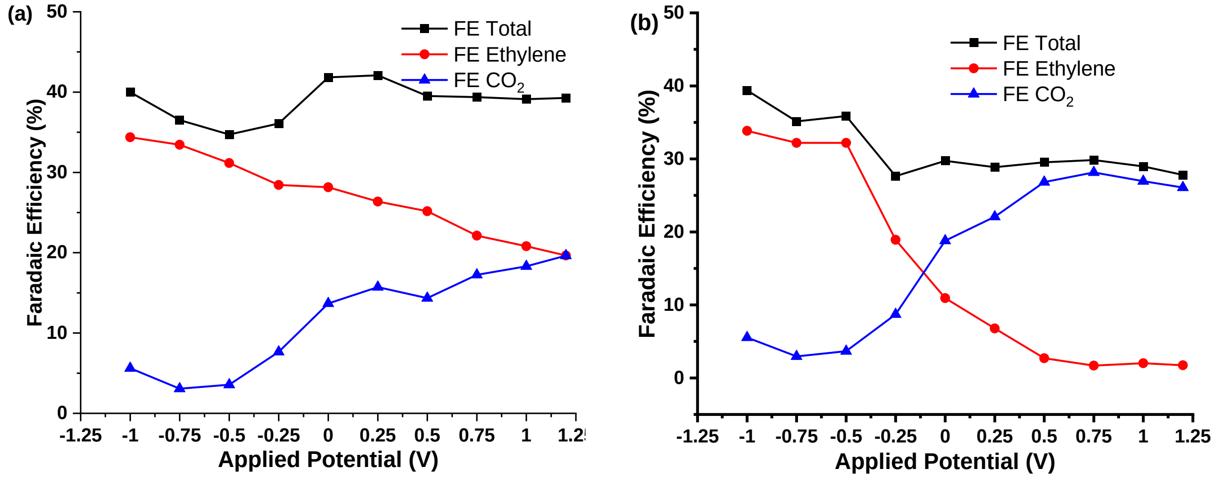

Supplementary Figure 8. Faradaic efficiency calculated during the chronoamperometric measurements at 850°C for (a) point 1 and (b) point 2 as indicated in Figure 2i. Black, red, and blue color curves indicate the Fes towards total, Ethyl, and CO2. The unaccounted current is attributed to water due to difficulty in quantifying water associated with condensation.

Supplementary Figure 9

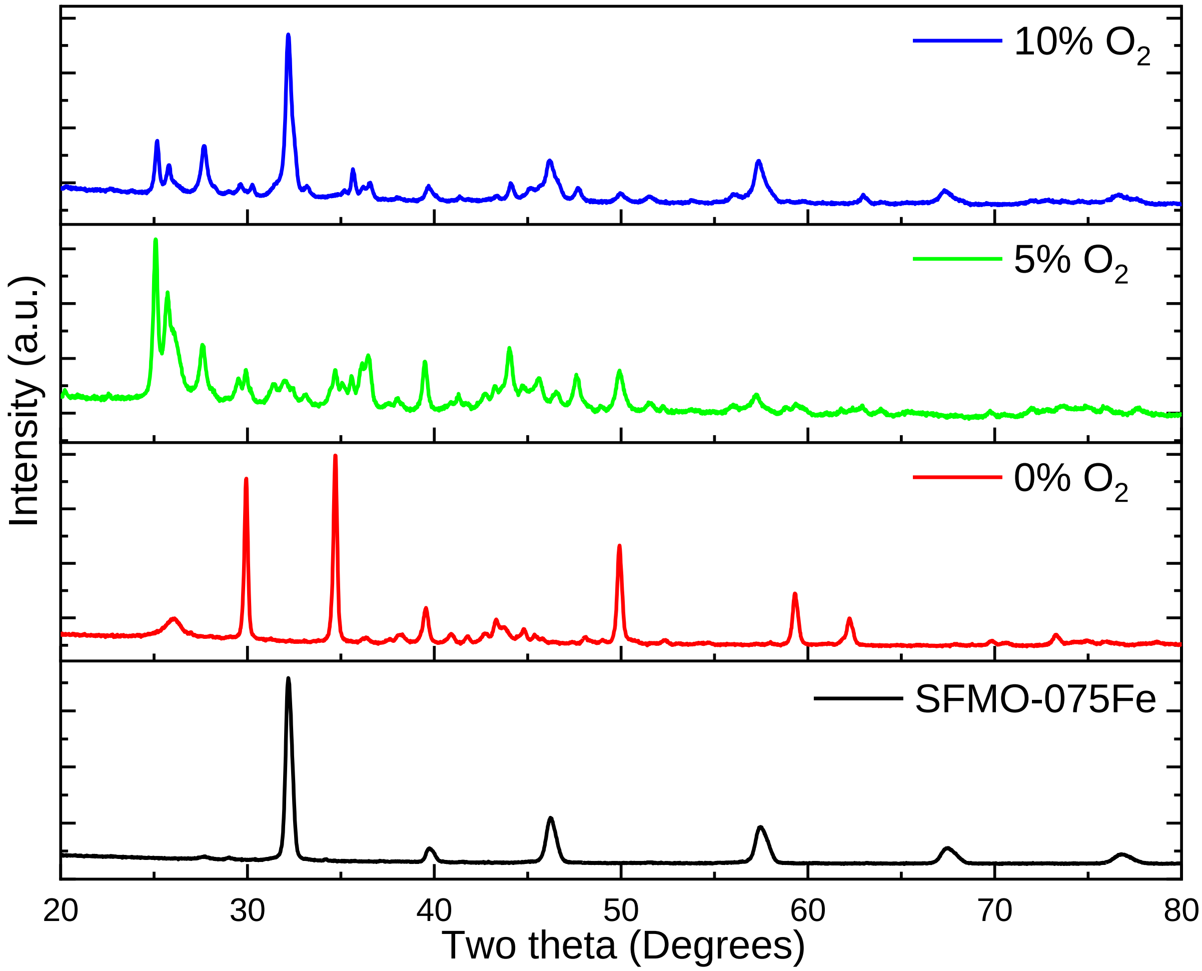

Supplementary Figure 9. PXRD patterns obtained for as-prepared SFMO-075Fe (black), after exposure to various methane to oxygen mixtures during temperature programmed oxidation measurements, red – 100% CH_4:0% O_2, green - 95% CH_4:5% O_2, and blue – 90% CH_4:10% O_2.

Supplementary Figure 10

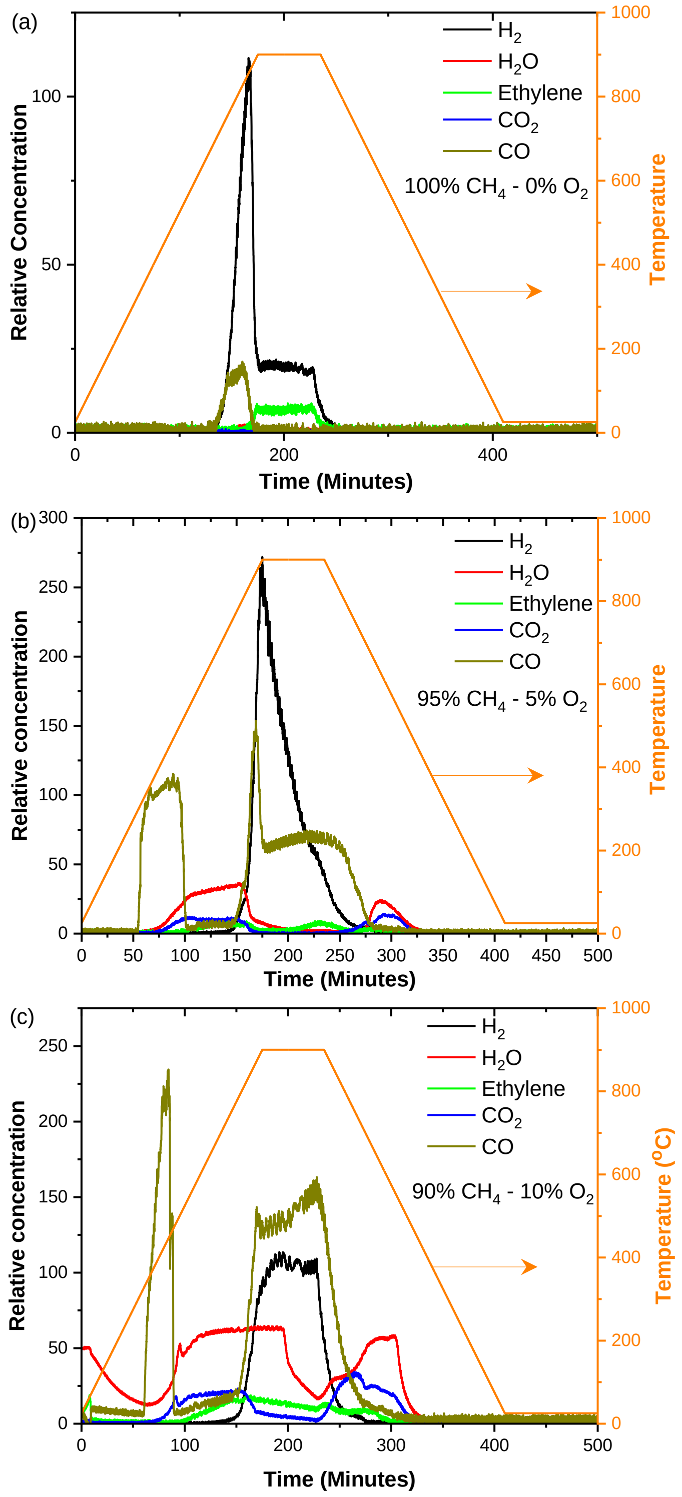

Supplementary Figure 10. Mass spectra results obtained during temperature programmed oxidation measurements on SFMO-075Fe samples under various CH_4 to O_2 ratios (a) 100% CH_4, (b) 95% CH_4:5% O_2, and (c) 90% CH_4:10% O_2. Black, red, and green curves indicate H_2, H_2O and ethylene while blue and dark yellow colors indicate CO_2 and CO respectively.

Supplementary Figure 11

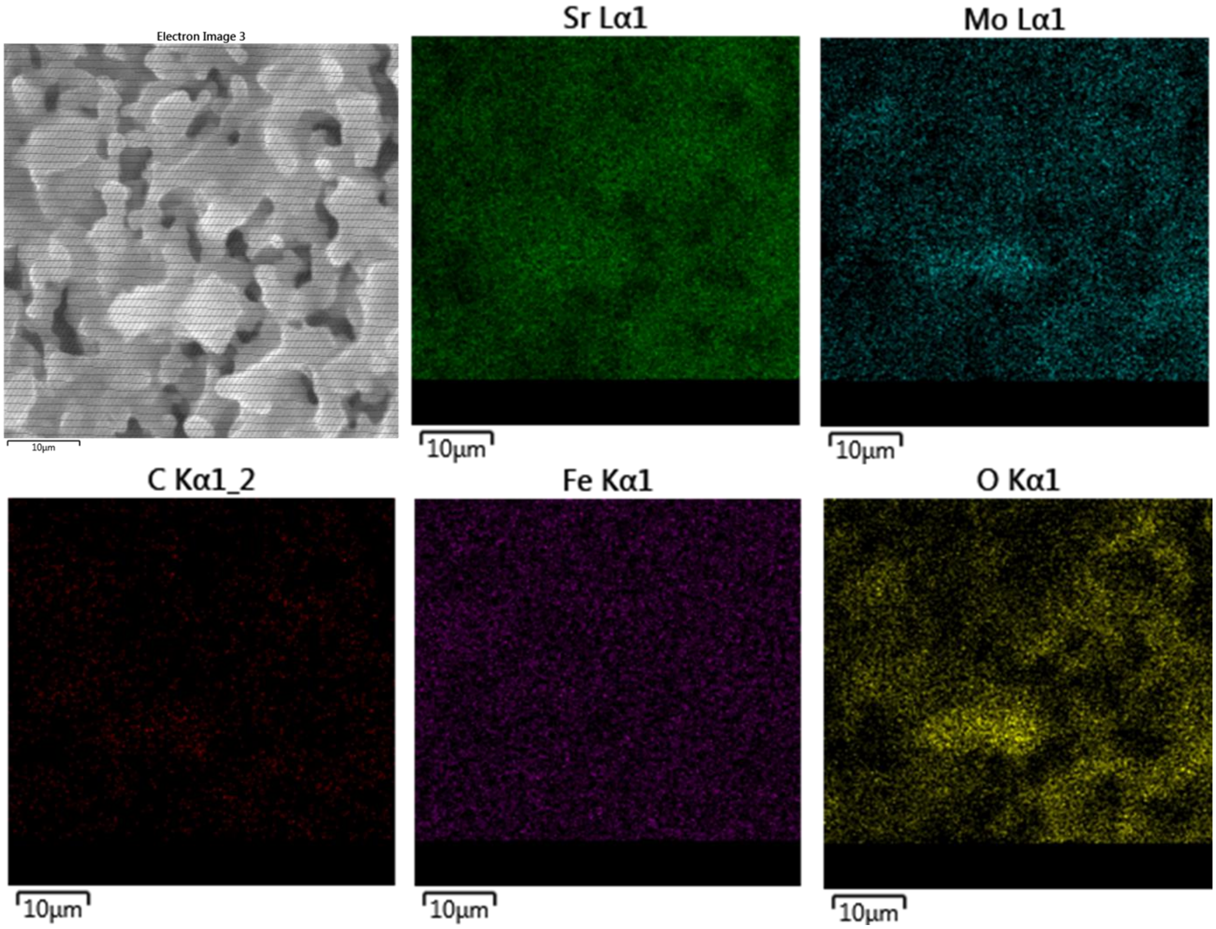

Supplementary Figure 11. EDX elemental mapping obtained for the as-prepared SFMO-075Fe that shows an uniform distribution of Sr, Fe, Mo and O on the material.

Supplementary Figure 12

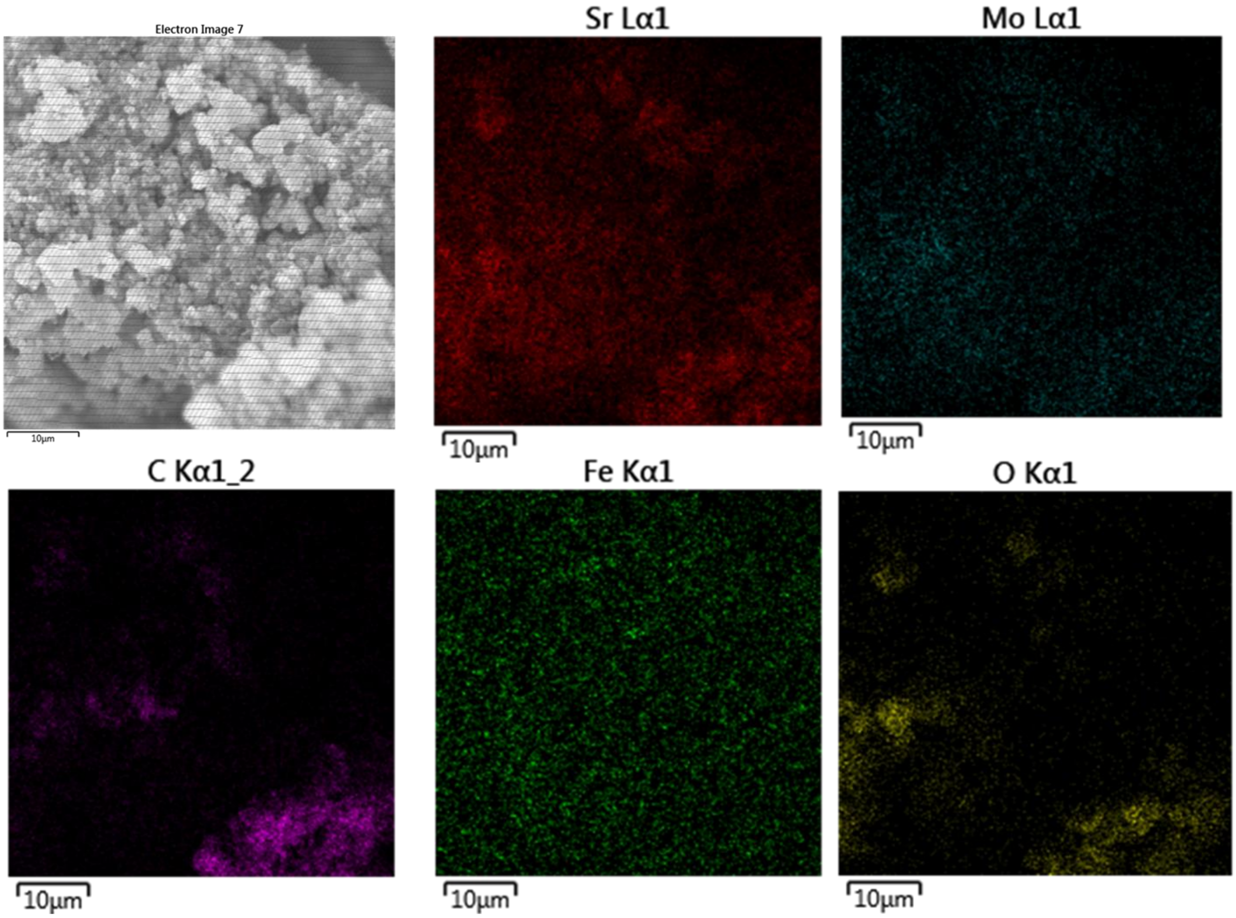

Supplementary Figure 12. EDX elemental mapping obtained for the methane treated SFMO-075Fe that shows an increase in carbon content especially on the powder materials on top layers. This is in accordance with an increase in carbon content from 15 at% to 84 at%.
